# Supplementary material for: Symptomatic fever management in children: A systematic review of national and international guidelines
Source: PLoS One. 2021 Jun 17;16(6):e0245815. doi: 10.1371/journal.pone.0245815 (PMC8211223; doi:10.1371/journal.pone.0245815)
Supplement: Attachment — Submitted filename: Response to Reviewers_18052021.docx [file pone.0245815.s003.docx]

| **Reviewer comment** | **Author response** |
| --- | --- |
| Abstract Page 2. Methods. Line 26. Please, include coverage dates (e.g. “from January 1990 to September 2020). | Thank you, included coverage dates Page 2. Methods. Line 27. |
| Page 2. Systematic Review Registration. Line 40. Please, provide registration information for the review (e.g. review protocol), including register name and registration number, or state that the review was not registered. Thank you. | Thank you, stated that the review was not registered. Page 2. Line 41. |
| Methods Page 3. Line 60. Provide registration information for the review (e.g. review protocol), including register name and registration number, or state that the review was not registered. Thank you. | Thank you, stated that the review was not registered. Page 3. Line 61. |
| Page 3. Line 30. Please, clarify if a reporting guideline (such as PRISMA statement) was used to report the methods and results, and include a populated checklist as a new Additional file. Thank you. | Thank you, Page 2 line 30?  We have added the statement to the abstract. Page 2. Line 31. A populated checklist was already submitted but we will resubmit. |
| Page 4. Selection of guidelines. Line 78. Please, state the process was used for selecting studies (such as two independent reviewers) through each phase of the review (that is, screening, eligibility and inclusion in the completed review). | Thank you, we have stated the process for selecting studies was performed by one author. Page 4. Lines 80-82 |
| Page 4. Data extraction. Lines 89-93. Please, describe the method of extracting data from reports (such as piloting forms, done independently, in duplicate), any processes for obtaining and confirming data from guideline developers. Thank you. | Thank you, we have stated the data was extracted by one author into an excel table. Page 4. Line 94. |
| Discussion Provide a general interpretation of the results in the context of other evidence (see comment above) (example ref. 8) | Thank you, we have provide a general interpretation of the results in the context of other evidence. Page 19. Line 183-186 |
| 1. Please ensure that your manuscript meets PLOS ONE's style requirements, including those for file naming. The PLOS ONE style templates can be found at  <https://journals.plos.org/plosone/s/file?id=wjVg/PLOSOne_formatting_sample_main_body.pdf> and  [https://journals.plos.org/plosone/s/file?id=ba62/PLOSOne_formatting_sample_title_authors_affiliations.pdf](about:blank) | Thank you, we have formatted our manuscript according to the PLOS ONE guidelines. |
| 2. Please include captions for your Supporting Information files at the end of your manuscript, and update any in-text citations to match accordingly. Please see our Supporting Information guidelines for more information: [http://journals.plos.org/plosone/s/supporting-information](about:blank). | Thank you, we have included captions for our supporting Information and named the files according to the guidelines. Line 67/104/156/160) |
| 1. Introduction section of abstract could be improved. | Thank you, we have expanded it. Lines 24-28. |
| 2. Line number 218 / 219: 'We share this opinion...' This should be re-worded; I feel that 'opinion' should not be mentioned here | Thank you, we have reworded the sentence to exclude “opinion”. |
| 3. Line number 225-229: I'm not sure it is appropriate to advise on this as, again, not based on evidence. | Thank you, have changed to read: “We did not identify any studies on this”. Line 226/227 |
| 4. Line number 236-238: 'The initial small reduction...rather than distress'. I would reword this to something like 'may not be worth...' | Thank you, line 244? Have reworded to “may not be worth”. Line 244 |
| 1. It should be made clear if the guidelines analysed are destined for professionals or for parents. For example, the bibliographic citation 69 is referenced to a non-professional information page. This influences the content of the guidelines and the complexity of the recommendations given. If the review is mixed (health professionals and non-professionals) this should be clarified. | Thank you, we have clarified that the guidelines are for a mixed audience “All CPGs, whether intended for healthcare workers or parents”. Line 77/78 |
| 2. I would like to point out that the bibliography should be revised. All of it should be presented in English and with the URLs for access. | Thank you, we have presented all references in English, and where possible, with URL. |
